# Editorial on the Special Issue Titled “Pathology and Diagnosis of Gynecologic Diseases—2nd Edition”

**DOI:** 10.3390/diagnostics15030356

**Published:** 2025-02-04

**Authors:** Cinzia Giacometti

**Affiliations:** Pathology Unit, Ospedale P. Pederzoli, Peschiera del Garda, 37019 Verona, Italy; cinzia.giacometti@ospedalepederzoli.it

The pathology and diagnosis of gynecological diseases encompass many different organs (breasts, uterus, ovaries, salpinx, peritoneum, and placenta) and benign or malignant diseases.

As regards breast cancer, in the last few years, the use of checkpoint inhibitors in locally advanced or metastatic triple-negative breast cancer (TNBC) has widened the horizons of neoadjuvant chemotherapy. The use of these new drugs relies on the expression of the programmed death ligand 1 (PD-L1) [1], whose expression is tested via immunohistochemistry. As investigated by Ilieva and coworkers, the evaluation of PD-L1 expression is pivotal in the management of these patients, and its assessment after NACT seems to play an essential role in understanding the biological behavior of TNBC, mainly because it appears to be related to better pathological responses and the number of tumor-infiltrating lymphocytes (TILs) [2].

Endometrial lesions are particularly interesting in the vast scenario of gynecological diseases.

Among benign lesions, adenomyosis and endometriosis, two distinct gynecological disorders characterized by the ectopic growth of endometrial tissue, are particularly important, as they may cause significant discomfort, pain, and infertility in affected women. In the last few years, NOTCH1 and CD117 have been investigated to better understand these entities [3,4]. Metodiev et al. highlighted the possible different origins of these two similar entities. They studied the presence of NOTCH1+ and CD117+ endometrial stem cells, finding intriguing differences in their expression in extra-uterine endometriotic lesions [5].

Endometrial malignancies pose a complex challenge in gynecologic settings due to their increasing incidence and mortality rates. Key risk factors, such as obesity, prolonged estrogen exposure, and metabolic disorders, highlight the urgent need for non-invasive, early diagnostic tools, as was presented in the review by Porcaro et al. [6]. The authors explored the role of DNA methylation as a potential biomarker for the early detection of endometrial cancer, noting that abnormal DNA methylation in the promoter regions of tumor-suppressor genes can result in gene silencing and cancer progression. The review emphasizes the promise of DNA methylation-based testing as a non-invasive alternative to traditional diagnostic methods, providing earlier detection, improved risk stratification, and more personalized treatment plans.

The rise in precision, patient-tailored medicine relies on investigating the molecular mechanisms involved in cancer progression. Various prognostic factors have been extensively studied, including hypoxia-regulated proteins (HIF-1α) [7] and glucose transporters [8], which have bene identified as prognostic markers in various cancers. In contrast, their role in endometrial cancer remains unclear. In the paper by Song and coworkers, the authors aimed to evaluate HIF-1α and GLUT-1 expression in endometrial cancer and correlate their expression with clinicopathological features. They found that a higher proportion of HIF-1α-positive immune cells was associated with high-grade endometrial cancers and diffuse GLUT-1 expression patterns, suggesting a potential role for HIF-1α as a prognostic marker [9].

Many studies have investigated the impact of positive peritoneal cytology on the survival of endometrial cancer patients, even in the early stages, among more traditional prognostic factors such as stage and histologic grade. Even if the most recent FIGO stage eliminated it [10,11], the significance of positive peritoneal cytology remains a topic of debate, as was concluded in the paper by Fegus et al. [12].

Uterine malignancies also encompass extremely rare entities. Leiomyosarcoma arises from the myometrium, and when undifferentiated, it displays a high-grade or undifferentiated tumor, which can harbor heterologous elements. The diagnostic difficulties of assessing the correct diagnosis of a relapse of uterine leiomyosarcoma often rely on the absence of differentiated areas or the presence of heterologous elements in the previous tumors [13]. In the series presented by Kim et al., most relapsed leiomyosarcomas demonstrated dedifferentiated areas and heterologous components, while the primitive tumors were mainly conventional leiomyosarcomas [14].

Uterine mesenchymal tumors represent a diverse group of tumors that exhibit a wide range of morphologic, immunohistochemical, and molecular profiles; they are also associated with various clinical behaviors. In recent years, there has been an increasing classification of these tumors based on their underlying molecular alterations, resulting in the more accurate differentiation of diagnostic entities. This group includes perivascular epithelioid cell tumors (PEComas). PEComas of the female reproductive tract have primarily been documented in case reports due to their clinical rarity. The limited incidence rates and clinical case data obstruct our thorough understanding of this disease [15,16]. In their “Interesting Images” article, Badlaeva and colleagues presented a rare case of PEComa raised in the pelvic region of a young girl [17].

Gynecological malignancies can also develop in the fallopian tubes. Serous tubal intraepithelial carcinoma (STIC) is an early-stage cancerous lesion in the tubal fimbriae. STIC is recognized as a precursor to many high-grade serous carcinoma (HGSC) cases, which originate in the fallopian tubes. Its development is often associated with mutations in the TP53 gene, resulting in the formation of a p53 signature—an early abnormality that may advance to HGSC. This signature is more prevalent in BRCA mutation carriers, accounting for the higher STIC incidence in this group [18]. In their review, Luvero et al. assessed the literature from 2016 to 2023 regarding the incidence of serous tubal intraepithelial carcinoma in patients (both BRCA-positive and BRCA-negative) undergoing preventive salpingo-oophorectomy. They concluded that the overall incidence of STIC among all women included in the studies was approximately 7%, while the incidence in BRCA-mutated women was approximately 6%. Moreover, the presence of the p53 signature was significantly linked to the occurrence of STIC [19].

HPV infection of the cervix can still be challenging, even in the era of HPV vaccination. The battle against HPV infectious goes through the adoption of updated and efficient follow-up strategies, as was well explained by Kaya Terzi and Yulek in their article. The application of co-testing (pap smear + HPV DNA) can reduce unnecessary interventions (such as pap smear repetition, colposcopy, and biopsy) [20,21]. The implementation of non-invasive strategies includes the possibility of testing miRNA as biomarkers for cervical dysplasia, as was described by Wittenborn et al. in their paper [22]. Challenges may also be encountered when dealing with apparently synchronous HPV-related lesions, as reported by Wegscheider et al., who described the concomitant presence of an HPV-associated invasive squamous cell carcinoma of the uterine cervix with the unexpected detection of an HPV16-positive high-grade squamous intraepithelial lesion of the fimbria of the right fallopian tube. In these cases, the “field-effect” theory (HPV infection resulting in the concomitant development of primary squamous cell lesions in various sites of the female genital tract) seems to be the most reliable [23,24].

Notably, in gynecological pathology, tissue artifacts may also play a role in defining a diagnosis. A benign lesion can be misdiagnosed as malignant due to foci of vascular pseudo-invasion, myometrial clefts, and tumor cells in the lumen of the cervix, on the serosa, and in the tubal lumen. These artifacts are relatively frequent (up to one-third of the hysterectomy specimens studied) and must be kept in mind to avoid overdiagnosis [25].

The compilation of the articles in this Special Issue on gynecologic pathology covers a wide range of research, reflecting the richness of this field. The studies adopted different methodologies, including observational approaches, reviews of the literature, and case studies. It is worth noting that the articles published in this Special Issue often address practical issues that can be encountered in clinical practice. Sharing this knowledge offers the readers a chance to gain a broader understanding of the research field of gynecologic pathology and its application in daily clinical routine.
